# KritiSamhita: A machine learning dataset of South Indian classical music audio clips with tonic classification

**DOI:** 10.1016/j.dib.2024.110730

**Published:** 2024-07-09

**Authors:** Samhita Konduri, Kriti V. Pendyala, Vishnu S. Pendyala

**Affiliations:** a Palo Alto High School, 50 Embarcadero Road, Palo Alto, CA 94301, USA; b University Preparatory Academy, 2315 Canoas Garden Ave, San Jose, CA 95125, USA; c Department of Applied Data Science, San Jose State University, One Washington Square, San Jose, CA 95192, USA

**Keywords:** Indian classical music, Music tonic, Raw audio snippets, Machine learning

## Abstract

There are currently a limited number of Indian classical music datasets, especially those large enough and with useful annotations, particularly the subtler ones, such as the tonic, for training classification or prediction models. The dataset described in this paper is created with useful tonic annotations, to fill this gap. The tonic pitch, or base pitch, plays an important role in music, so much so that it is sometimes called the keynote. The vocalists and the accompanying instrumental ensemble are fine-tuned to this keynote to render the composition. The first and second authors of this paper, who are vocalists themselves, recorded songs in four different tonics: F#, G, G#, and A. Using the Python library pydub, each 3+ minute song was segmented into 20-second snippets, including the remainder as a separate snippet. The raw audio snippet data is available in folders separated by tonic, and a directory contains each snippet's file path and tonic. This dataset can be reused for tonic classification work in the future, as well as for training other automated systems targeting higher-level attributes of ICM, such as melodic framework, as a tonic can be the basis for them all.

## Highlights

• This dataset aids in the training of machine learning models for the task of retrieving music based on the subtler aspect of tonic, which is a useful and difficult application.
• The results from experiments using this dataset can provide several valuable insights, particularly in dealing with finer-grained attributes of music such as the tonic.

Specifications Table

**Table:** 

Subject	Artificial Intelligence and Multimedia
Specific Subject Area	The tonic pitch plays a foundational role in Indian Classical Music (ICM), where, unlike Western music, the lead artist chooses a tonic that other artists attune to.
Data Types	Raw (Audio Files)
Data Collection	csv file - annotation directory mp3 files - audio snippets
Data Source Location	The audio files were recorded by the first and second authors, using QuickTime Player on macOS and the Recorder application on a Windows laptop, respectively. All snippets contain a sole vocalist singing, recorded with a drone in the background, playing a specific tonic. Google Colab was used to break up the 20-second snippets, using the Python library pydub. These snippets were stored on Google Colab and then uploaded to the Mendeley Data repository. In total, 4 tonic bins are represented by the dataset: F#, G, G#, and A. The dataset contains 300 F#, 207 G, 240 G#, and 280 A snippets.
Data Accessibility	Audio snippets recorded in Palo Alto and San Jose, California, USA by the first and second authors.
	Repository name: Mendeley Data [1]
	Data identification number: 10.17632/nkdm57hvw3.2
	Direct URL to data: https://data.mendeley.com/datasets/nkdm57hvw3/2
	Code: [2]
	Instructions for accessing these data: The dataset contains a CSV file with tonic annotations, as well as a ZIP file containing audio snippets, separated into four folders based on tonic.

## 1 Value of the Data

• This dataset is one of the few Indian classical music datasets that includes raw audio data, as well as tonic annotations, which are foundational for higher-level attributes. In addition, these data contain snippets covering four different tonics, ranging from F# to A, which are the most commonly used tonics.
• These data were recorded by artists who have varying degrees of knowledge in Carnatic music, making their songs and alignment to the tonic more reputable.
• This dataset can be reused by other researchers to train their classification/prediction models and other automated systems to predict and leverage tonic. These data can also be valuable in helping new or experienced music learners test their pitch and find which tonic works best for them.
• Outside Machine Learning, this dataset can be used by music students to become more skilled at identifying tonic and staying in tune. It can be used by musicologists, specifically in systematic musicology, to learn more about how tonic affects how different songs are perceived. It can be used in psychology to understand how the tonic of a song can affect the listeners’ mood.

## 2 Background

KritiSamhita means a collection of musical compositions and is the name of the dataset being described. The term “kriti” stands for “creation” or “work” in Sanskrit and designates the primary method of musical composition in Carnatic music. It is a song with a particular arrangement that is a main feature in Carnatic music performances. Kriti is the fundamental unit of a Carnatic music performance in terms of musical form. A conventional kriti usually consists of three sections. First is Pallavi, which is the main refrain. It is analogous to a chorus in Western music. Second is Anupallavi. It is the second verse and is optional. The song's last and longest verse is called Charanam. As part of a lengthy heritage, Kriti serves as the foundational repertoire for the majority of Carnatic musicians who acquire it from their tutors.

The Sanskrit word, Samhita means a collection and expresses the idea of things being compiled or assembled following a particular methodology. KritiSamhita is therefore a systematic collection of vocal compositions that the dataset represents, classified by the music tonics of the compositions.

Tonic, also known as *shruti*, is an aspect of ICM representing the base note, which can vary based on an artist's choice [3]. Whereas Western music, based on harmony, does not require a single tonic note, ICM artists usually use a drone playing the tonic note in the background [4]. A survey of various approaches for tonic identification [5] shows that it is a growing area of research. Previous work has been done to account for a tonic with entropy thresholds [6] and manual annotations [7]. Other work used chromagram data with the specific melodic framework to determine tonic [8]. Previous work determined that tonic is usually chosen by an artist using a trial-and-error process [9]. There is potential to use automated systems in this area. Beyond automated systems for tonic, because tonic is foundational for other higher-level attributes, it has been recognized that tonic information is necessary for other types of automated systems. For example, the tonic was used as a feature for music genre classification [10]. In the large problem of melodic framework identification, studies have used tonic annotations in their datasets for normalizing pitch [11]. There have also been attempts to create an automated Indian Classical Music tutor using similar ideas [12] and converting from one melodic framework to another using a CycleGAN [13].

Currently, there are a limited number of datasets relevant to Indian Classical Music (ICM). The authors of this paper used one such dataset to use machine learning techniques to discern finer-grained attributes of music specifically about the vocal artist [14]. This work creates a large dataset classified on another finer-grained aspect, the tonic. The authors used this dataset to train classification models and detect the tonic of music snippets [15].

## 3 Data Description

**Fig. 1:**
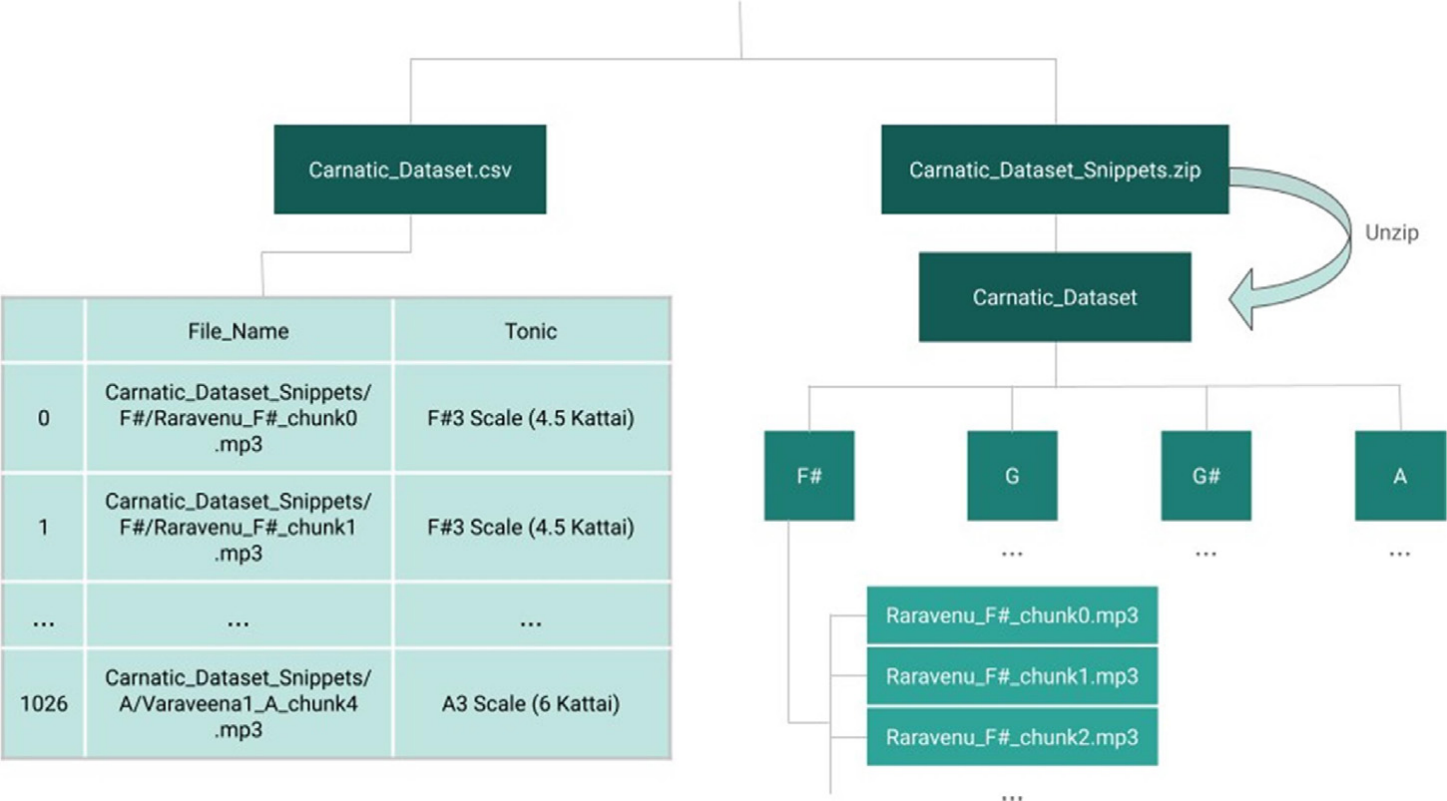
Layout of the dataset. The CSV file functions as a directory, with the file name and tonic annotations.

The dataset contains two main files: “Carnatic Dataset Snippets.zip”, a ZIP file containing the 20-second audio snippets, and “Carnatic Dataset.csv”, a CSV file containing the metadata and tonic annotations. Fig. 1 graphically represents the layout of the dataset, explained below.

Specifically, unzipping “Carnatic Dataset Snippets.zip” extracts a folder called “Carnatic Dataset Snippets”. This contains 4 folders, for each of the 4 tonic bins represented: “F#”, “G”, “G#”, and “A”. In each tonic folder, there are mp3 files for each snippet, containing the raw audio data. The snippets are named in the following format: {songName}_{tonic}_chunk{number} .mp3. For example, the song *Raravenu*, recorded in the F# tonic, was broken up into 14 snippets. The first 13 are exactly 20 seconds long, and the last one is the remaining part of the song (which is less than or equal to 20 seconds). The first chunk or snippet is named “Raravenu F# chunk0.mp3”, and the remaining snippets are similarly named, with the chunk number ranging from 0 to 13. The same formula for naming is used across songs and tonics.

The CSV file functions as a dictionary for this dataset, containing two columns: “File Name” and “Tonic”. The column “File Name” contains the full file path for each snippet, starting from the folder that contains the main dataset folder Carnatic Dataset. The column “Tonic” contains the tonic annotations associated with each snippet. The tonic annotations are named following the following format: {Tonic} Scale ({Conversion to South Indian Classical Music} Kattai). *Kattai* is the measurement for tonic in South Indian Classical Music, measured with numbers in increments of 0.5. A tonic of F# corresponds to a *kattai* of 4.5, G to 5, G# to 5.5, and A to 6. For example, the snippets of *Raravenu* in the example above would be annotated with “F# Scale (4.5 Kattai)” in the CSV file. In total, the dataset contains 300 snippets in F#, 207 in G, 240 in G#, and 280 in A.

## 4 Experimental Design, Materials, and Methods

First, all songs were recorded by the first and second authors of the paper, with a drone in the background as the only accompaniment. The drone was playing a fixed tonic note, which the vocalist used as their base note while singing. The songs recorded by the first author were recorded using QuickTime Player on macOS, with a built-in MacBook Air Microphone, with a default sampling rate of 44100 Hz. They were recorded in an emptier room of about 500 square feet, with a slightly higher chance for an echo or reverb in the recording. The drones were played using the “Dhwani Tanpura” app on the Apple App Store. The songs were converted to MP3 format using the online site CloudConvert [16]. The songs recorded by the second author were recorded using the Sound Recorder application and build-in microphone on a Windows laptop, with a default sampling rate of 48000 Hz. They were recorded in a smaller room with other large furniture inside, limiting the chance of an echo in the recordings. The drones were played using videos from the YouTube channel YourTanpura [18]. The songs were converted to MP3 format using the online site FreeConvert [17].

Then, on Google Colaboratory, using the Python library pydub, the recordings, most over 3 minutes long, were separated into 20-second snippets. A final snippet was saved with the last seconds of the song, the remainder after uniform 20-second snippets were saved. Along with the segmentation, the tonic and file name were written to a CSV file. Finally, the dataset was compressed to a ZIP file. The program installed and used the pydub library, and mounted Google Drive to access and save files. The Python os module was also used to iterate through a list of songs in each tonic. Finally, the library Pandas was used to save tonic annotations as a dataframe, which was finally downloaded as a CSV file.

The Jupyter Notebook with all of the code used to format and create the dataset can be accessed on Github [2].

## Limitations

This dataset entirely contains recordings from two vocalists, both with female voices, which reduced the diversity of samples, introducing some bias. Additionally, most vocalists can only comfortably sing in 2-3 tonics, so only having two vocalists recording the dataset limited the tonics covered. The dataset only spans 4 tonics, whereas there are over 20 different tonics that artists can use. Finally, the two artists who recorded this dataset have slightly different voices and rendition styles, which could lead to discrepancies in the data, and low diversity of voice types.

## Ethics Statement

The authors have read and followed the ethical requirements for publication in Data in Brief and confirm that the current work does not involve human subjects, animal experiments, or any data collected from social media platforms.

## CRediT authorship contribution statement

**Samhita Konduri:** Methodology, Software, Validation, Formal analysis, Investigation, Resources, Data curation, Writing – original draft, Visualization. **Kriti V. Pendyala:** Investigation, Data curation, Resources. **Vishnu S. Pendyala:** Conceptualization, Methodology, Formal analysis, Writing – review & editing, Supervision, Project administration, Funding acquisition.

## Data Availability

KritiSamhita: South Indian Music Tonic Recognition Dataset (Audio) (Original data) (Mendeley Data).
